# Adolescent pregnancy and linear growth of infants: a birth cohort study in rural Ethiopia

**DOI:** 10.1186/s12937-019-0448-0

**Published:** 2019-04-02

**Authors:** Abdulhalik Workicho, Tefera Belachew, Alemayehu Argaw, Shibani Ghosh, Meghan Kershaw, Carl Lachat, Patrick Kolsteren

**Affiliations:** 1 Department of Epidemiology, Institute of Health, Jimma University, P.O.Box 378, Jimma, Ethiopia; 2 Department of Population and Family Health, Institute of Health, Jimma University, P.O.Box 378, Jimma, Ethiopia; 3 Department of Food Technology, Safety and Health, Ghent University, Coupure links 653, B 9000 Ghent, Belgium; 4 Friedman School of Nutrition Science and Policy, Tufts University, 150 Harrison Avenue, Boston, MA USA

**Keywords:** Maternal age, Adolescent pregnancy, Linear growth, Infant growth

## Abstract

**Background:**

Evidences indicate that the risk of linear growth faltering is higher among children born from young mothers. Although such findings have been documented in various studies, they mainly originate from cross-sectional data and demographic and health surveys which are not designed to capture the growth trajectories of the same group of children. This study aimed to assess the association between young maternal age and linear growth of infants using data from a birth cohort study in Ethiopia.

**Methods:**

A total of 1423 mother-infant pairs, from a birth cohort study in rural Ethiopia were included in this study. They were followed for five time points, with three months interval until the infants were 12 months old. However, the analysis was based on 1378 subjects with at least one additional follow-up measurement to the baseline. A team of data collectors including nurses collected questionnaire based data and anthropometric measurements from the dyads. We fitted linear mixed-effects model with random intercept and random slope to determine associations of young maternal age and linear growth of infants over the follow-up period after adjusting for potential confounders.

**Results:**

Overall, 27.2% of the mothers were adolescents (15–19 years) and the mean ± SD age of the mothers was 20 ± 2 years. Infant Length for Age Z score (LAZ) at birth was negatively associated with maternal age of 15–19 years (β = − 0.24, *P* = 0.032). However, young maternal age had no significant association with linear growth of the infants over the follow-up time (*P* = 0.105). Linear growth of infants was associated positively with improved maternal education and iron-folate intake during pregnancy and negatively with infant illness (*P* < 0.05).

**Conclusion:**

Young maternal age had a significant negative association with LAZ score of infants at birth while its association over time was not influential on their linear growth. The fact that wide spread socio economic and environmental inequalities exist among mothers of all ages may have contributed to the non-significant association between young maternal age and linear growth faltering of infants. This leaves an opportunity to develop comprehensive interventions targeting for the infants to attain optimal catch-up growth.

**Electronic supplementary material:**

The online version of this article (10.1186/s12937-019-0448-0) contains supplementary material, which is available to authorized users.

## Introduction

Inadequate growth among infants is commonly expressed as a failure in linear growth with low length-for-age Z-score (LAZ) [1]. Failure to reach optimal growth potential is an important indicator of chronic under-nutrition [2] and increases the risk of morbidity and mortality in infants [1, 3–6]. For many children, failure in linear growth sets on before birth due to poor maternal nutrition and continues deteriorating reaching a plateau approximately at the age of two years [7]. Although it is hypothesized to result mainly from infant under nutrition, the deterioration is also determined by fetal developmental insults, infection and other environmental factors. Comprehensive approaches addressing vulnerable periods of the life course are required instead of interventions targeting solely infant-toddler nutrition in promoting optimal child growth in low-and middle-income countries (LMICs) [1, 5].

A growing body of evidence indicates that the risk of linear growth faltering and other adverse neonatal and infant health outcomes are higher among children born from young mothers due to socio-economic, behavioural and biological disadvantages [8–11]. Poor maternal nutritional status during pregnancy, poor child caring practices, lower socio economic status (SES) and lower educational level are common among young mothers potentially affecting the growth and development of their infants negatively [12, 13]. Child caring practices are affected as younger mothers are more likely breastfeed for a shorter period of time and are behaviourally immature to attend to child needs [14, 15]. Younger mothers are also more likely to hold lower educational level which could adversely affect their knowledge and child caring practices [12]. Young mothers in LMICs are also physically not well developed as they are still growing themselves. In some cases, their nutritional need can also be in competition with that of their infants [2, 14, 16] thus affecting intrauterine growth and birth weight and subsequently post natal infant growth. Deprivations can lead to a vicious cycle and result in intergenerational continuation of malnutrition [17, 18].

Existing reviews indicate a substantial gap of evidence in understanding the net effect of young maternal age on neonatal and infant nutritional outcomes [19–21]. Moreover, there is a paucity of evidence regarding the tracking of growth patterns of infants from young mothers in LMICs. Most findings originate from cross-sectional data and demographic and health surveys which are not designed to capture the growth trajectories of the same group of children [22]. We hypothesised young maternal age at birth is negatively associated with linear growth of their infants. We used data from a prospective birth cohort study, which followed the mother-infant pair for 12 months after birth to investigate the relationship between young maternal age and linear growth of infants. The results of this study are intended to inform policy and practice, with a focus on mitigating adverse nutritional consequences for infants, especially those who are born to young mothers.

## Methods and material

### Study setting and design

Data used for this study were obtained from the Empowering New Generation in Nutrition and Economic opportunities (ENGINE) birth cohort study, which was conducted from January 2014 to March 2016 in the Oromia Region of Ethiopia. The region was selected purposively being the largest in the country targeted by ENGINE with relatively high rates of stunting. Three districts, namely Goma, Woliso and Tiro Afeta, were further selected based on (i) an expected population of more than 3000 pregnant women to account for loss to follow up, (ii) geographical similarities in agro-ecology and agricultural production practices and (iii) proximity and accessibility. Administratively, each district was further divided into units called kebeles and all recruitment took place at the kebele level. A minimum sample size of 1281 subjects was required to detect a moderate effect size of 0.3 standard deviations (SDs) in infant linear growth over 12 months of follow up (equivalent to an effect size of 0.025 SDs in monthly changes of infant linear growth), assuming an auto correlation of 0.3, 80% statistical power, a one to three adolescent to adult pregnant women ratio, a type I error of 5% and a 30% attrition rate [23]. We therefore included a total of 1423 mothers from the original study who met the inclusion criteria for this study. Inclusion criteria were women of age 15–24 years with a singleton live birth without any congenital anomalies. The mother-infant pair was followed for 12 months after delivery. However, the analysis was based on 1378 subjects with at least one additional follow-up measurement to the baseline.

### Measurements

A team of trained data collectors including nurses followed the pair during the study period. There were five time points, each three months apart, at which measurements were taken. Data on household characteristics, socio-economic and demographic information, antenatal exposures and dietary information were collected using a structured, pre-tested, interviewer-administered questionnaire using Android tablet computer at recruitment. Infant length was measured in a recumbent position to the nearest 0.1 cm using a length board (Weigh and Measure LCC, USA) and birth weight was measured to the nearest 10 g using a digital weighing scale (SECA 876, Hannover, Germany) with cloths removed. Low birth weight was defined as birth weight < 2500 g. Maternal height was measured using a stadiometer (Weigh and Measure LCC, USA) to the nearest 0.1 cm with no shoes on and with the five points touching the vertical stand of the stadiometer. A wealth index variable was constructed using principal components analysis based on data on housing conditions, ownership of durable assets and availability of basic services [24]. Infant illness was measured by maternal reporting of symptoms like fever, cough, diarrhea or other symptoms. The outcome variable LAZ was generated using the WHO standards [25] over time, as a continuous variable.

### Data quality control

Important precautions have been undertaken in order to ensure quality of the data at various stages of the study. Enumerators and their supervisors were recruited based on their prior experiences of engaging in such large scale surveys, fluency in speaking Afan Oromo, familiarity in using electronic data collection tools and their academic backgrounds. Afterwards, adequate training was given on each item of the data collection tool and how to take all the measurements needed in the study using practical applications through role playing. A three days long pretesting was also conducted in order to understand any variations in administering questions and taking measurements among the enumerators before commencing actual data collection. Electronic data collection method was used to minimize errors in data collection and entry by using android tablet computers. The collected data was checked by the supervisors in regular basis before the data was sent to a centrally located server. Additionally, a data manager regularly checked quality of the data and took back mistakes and incomplete data to the field to be corrected. A research team from Jimma University also closely followed up for technical and administrative supports. Refreshment trainings on data quality were given on a regular basis during the two years of study period.

### Statistical analysis

We used linear mixed-effects model with random intercept child and random slope time to fit child linear growth curve (change in LAZ) over the study follow-up period. Fixed-effects in the model included LAZ at birth, maternal age, time of study follow-up, a quadratic term of time and the interaction term between maternal age and time that compares maternal age categories on the evolution of child LAZ (linear growth) over time. A quadratic term of time was considered in the model to capture the nonlinear change in the growth curve. The use of other possible models using polynomial and spline functions of time were also considered for a better fitting model by comparing the AIC and BIC estimates of model performance (Additional file 1: Estimates table for the linear, quadratic and cubic-spline models). An unstructured correlation matrix was chosen for the correlation among repeated LAZ measurements per child after comparison of models using other correlation matrices. We considered the use of additional covariates of child growth to maternal age including maternal education, wealth index, iron-folic acid intake and infant illness. Model building was performed through several steps and the selection of important covariates in the final model was decided based on results of the regression outputs and consideration of the literature. We also assessed for effect modification by checking the interactions between the different covariates on child LAZ whenever found to be relevant. We performed different regression diagnostics assessing models goodness of fit (normality and heteroscedasticity of the residuals at different levels), model specification and other numerical problems like multicollinearity, and the sensitivity of the findings to potential influential observation. All the analysis was performed using STATA version 14 (StataCorp, Texas, USA) and all the tests were two-sided with a statistical significance considered at *p* < 0.05. *P* values were adjusted for multiple testing of hypothesis using Benjamini-Hochberg method [26].

### Ethical issues

Ethical approval was granted from the Institutional Review Board of Jimma University in Ethiopia (RPGC/264/2013) and Tufts University in USA (Tufts Health Sciences Campus IRB reference number:11088) before commencement of the study. Informed consent was obtained from the participants after a detailed explanation of the objectives of the study. Data was registered and stored in a secured server and access to the data was upon permission of the principal investigators with personal identifiers removed. During the study women or infants who had health problems were referred to a nearby health facility to seek proper medical care.

## Results

**Fig. 1:**
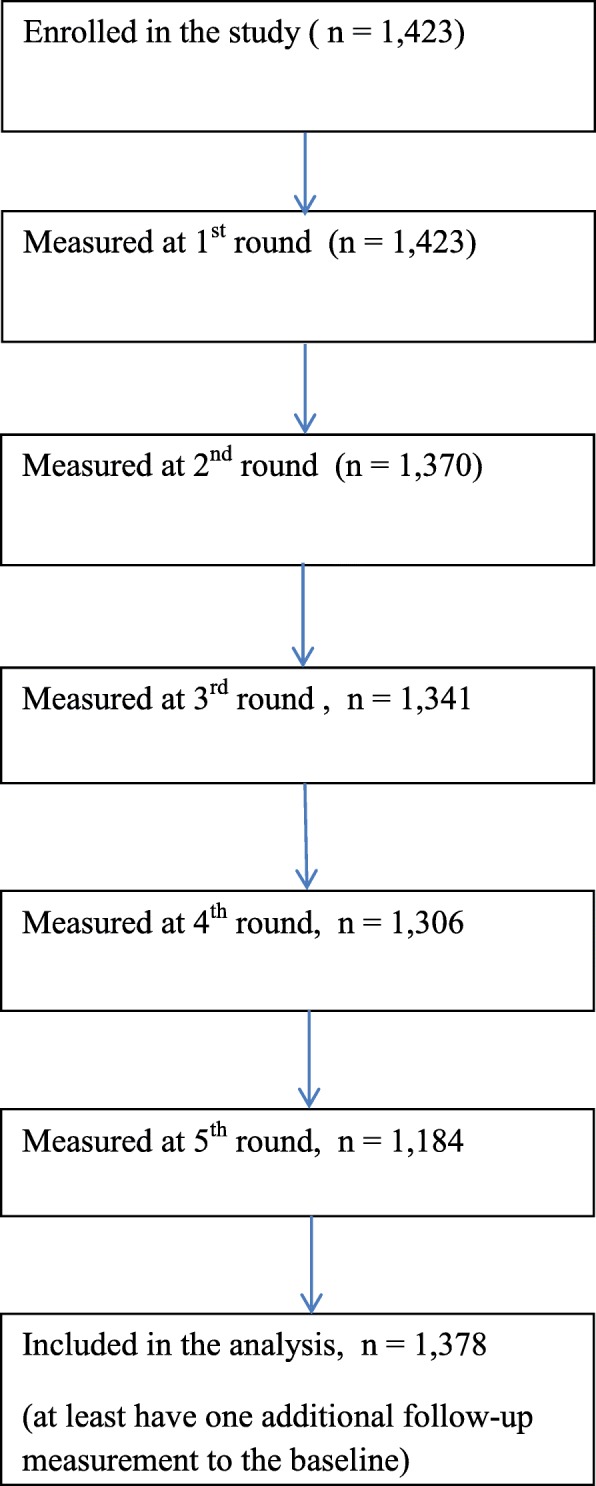
Flow diagram of follow up of study participants

A total of 1378 infants with at least one follow up measurement to the baseline were included in the analysis. Length was measured in all five rounds for 1184 (84%) of the infants and average number of measurements per child was 4.6. We have used the Strengthening the Reporting of Observational Studies in Epidemiology-nutritional Epidemiology (Additional file 2: STROBE-nut) [27] guideline to report findings in this manuscript. Figure 1 shows the follow up of study participants throughout the study period. Half (50.5%) of the infants were males and there was a significant variation by the level of maternal education among pregnant women who were 15–19 and 20–24 years of age (*P* < 0.001). The results of the descriptive analysis are presented in Table 1.

**Table 1:** Distribution of study variables by maternal age at delivery

Variables	Maternal age in years		
	15–19 (*n* = 387)	20–24 (*n* = 1036)	*p*-value
Maternal education			
no formal education	86 (22.2)	372 (35.9)	< 0.001*
primary	273 (70.5)	566 (54.6)	
secondary & above	28 (7.2)	98 (9.5)	
Wealth index			
Lowest	152 (39.3)	377 (36.4)	0.60
Middle	109 (28.2)	304 (29.3)	
Highest	126 (32.6)	355 (34.3)	
iron-folic acid intake, (> 90 days)	113 (29.2)	326 (31.5)	0.41
Illness in previous 2 weeks	223 (57.6)	647 (62.5)	0.09
infant sex, (male)	187 (48.3)	517 (49.9)	0.60
Mean(±SD) length (cm)			
At birth	49.32 (2.26)	49.53 (2.21)	
3rd month	60.52 (2.40)	60.87 (2.62)	
6th month	65.54 (2.68)	65.71 (2.71)	
9th month	69.12 (2.62)	68.92 (2.69)	
12th month	72.22 (2.53)	71.95 (2.65)	

*statistically significant difference at *p* < 0.05

**Fig. 2:**
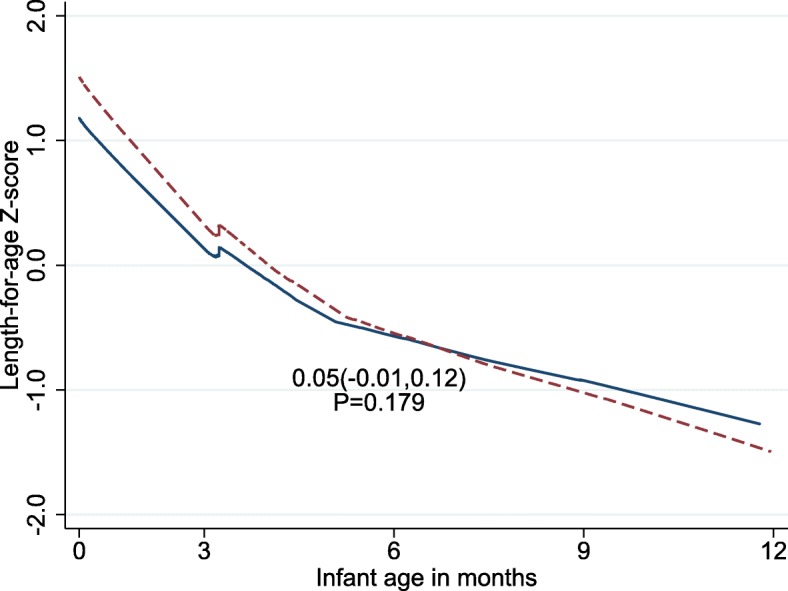
Linear growth of infants over 12 months from Mothers of 15–19 years old (---------) and Mothers of 20–24 years (______). Plotted values for the difference from baseline of the repeated measurements are estimated from linear mixed-effects model using random intercept child and random slope time with fixed effects including time, quadratic time, maternal age group, and timeXmaternal age group interactions adjusted for important covariates. The *P*-value is for group difference on monthly changes of LAZ score over time. β coefficient on monthly changes in LAZ was 0.05; *P* = 0.179

**Fig. 3:**
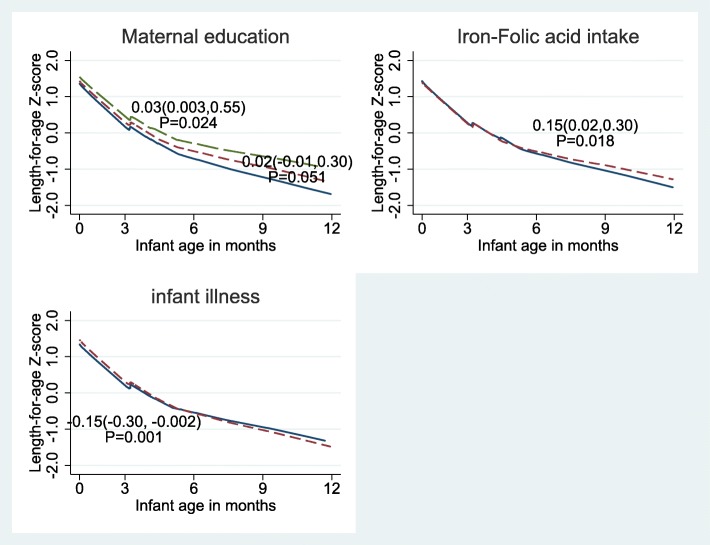
linear growth of infants by maternal and infant characteristics: Linear growth and maternal educational status; no formal education (____), primary (---------) and secondary and above (— — —), linear growth and iron and folic acid intake; < 90 days (--------) and ≥ 90 days (_____) and linear growth and infant illness; No (____) and Yes (-------). *P*-values are for group difference on monthly changes of LAZ score over time. β (95% CI) on monthly changes in LAZ were 0.03 (0.003,0.55); *P* = 0.024 for Maternal educational status of secondary and above, 0.02 (− 0.01,0.30); *P* = 0.051for primary maternal educational status, 0.15 (0.02,0.30); *P* = 0.018for iron-folic acid intake, − 0.15 (− 0.30, − 0.002); *P* = 0.001 for infant illness

Figure 2 presents the linear growth curve of infants over the 12 months follow-up period by maternal age category and adjusted for important covariates. Infants from adolescent mothers tend to have lower LAZ score compared to those born to mothers older than 20 years during the first 8 months of follow up. These infants do catch-up in their growth to surpass their counter parts after 8 months of age, but the association of young maternal age with the infant linear growth was not significant. On the other hand, both positive and negative associations were exhibited with some maternal and child characteristics and linear growth of the infants as displayed in Fig. 3.

Table 2 shows that infant’s LAZ score at birth was negatively associated with maternal age of 15–19 years (β = − 0.24, *P* = 0.032) after controlling for other covariates. However, young maternal age had no significant association with linear growth of the infants over the follow up time (*P* = 0.179). Maternal educational level of secondary and above (β = 0.03, *P* = 0.024) was associated with increase in linear growth of the infants over the follow-up period. Similarly, maternal intake of iron folate supplements for 90 days or more was also associated with an increase in LAZ score over time (β = 0.15, *P* = 0.018). Children from mothers of better education and with iron-folic acid supplementation of 90 days and more seem to have a better linear growth. On the contrary, infants who had illness had a lower LAZ score (β = − 0.15; *P* = 0.001). Figure 3 shows linear growth of infants by selected maternal and infant characteristics.

**Table 2:** Parameter estimates from linear mixed effects model predicting linear growth of infants

Fixed effects (*n* = 1378)	Coefficient (β)	SE	*P*
Intercept	1.38	0.143	< 0.001*
Infant age (Time)	0.14	0.029	< 0.001*
Quadratic time effect (Time*Time)	−0.01	0.002	< 0.001*
Maternal age (15–19 years)	− 0.24	0.128	0.032
Maternal age*Time	0.05	0.034	0.179
LAZ at birth	0.38	0.029	< 0.001*
Maternal education (ref = no formal education)			
Primary	0.05	0.077	0.518
Primary*Time	0.02	0.007	0.051
≥ Secondary	0.10	0.135	0.417
≥ Secondary*Time	0.03	0.013	0.024
Wealth index (ref = low)			
Middle	0.21	0.084	0.004*
Middle*Time	−0.05	0.008	0.485
High	0.13	0.084	0.018
High*Time	0.01	0.008	0.245
Iron-folate supplement (≥90 days)	−0.07	0.074	0.305
Iron-folate*Time	0.15	0.007	0.018
Illness in 2 weeks (Yes)	0.05	0.070	0.495
Illness in 2 weeks *Time	−0.15	0.006	0.001*
Random effects			
Variance of random intercept	0.004	0.006	
Variance of random slope	0.913	0.063	
Covariance of random intercept and slope	−0.033	0.005	
Variance of residuals	0.402	0.011	

Restricted maximum likelihood estimation (REML) was used to estimate the parameters, *SE* standard error, *Statistical significance after adjustment for multiple testing using Benjamini-Hochberg method

## Discussion

The present study examined the association between young maternal age at delivery and linear growth of infants. LAZ score at birth of infants was negatively associated with young maternal age while infant illness is negatively associated with infant linear growth during the 12 month follow-up period. Studies of the effects of young maternal age on infant linear growth generally reported that the findings vary by context attributing relative roles of biological, socioeconomic and child care factors for the differences [8, 10, 28]. In our analysis, we observed that LAZ score of infants of young mothers was lower compared to infants of older mothers at birth. Nutritional and biological disadvantages are higher in young mothers, which interfere with the development of the fetus affecting fetal growth. Reports from the same cohort and other studies have demonstrated that young maternal age at delivery is associated with adverse birth outcomes, including childhood stunting, smaller birth length, small for gestational age and other adverse neonatal and infant health problems [9, 22, 29].

Unlike the association with the LAZ score at birth, the interaction of young maternal age with time was not statistically significant. Linear growth did not vary significantly by maternal age among the infants during the follow up period. Maternal youth, through its effect on nutritional, socio economic and behavioral factors could impair fetal development and hence impair linear growth of infants [10, 28, 29]. Although it is not a complete catch-up, where trans-generational catch up is still required, these developmental insults can be averted during early childhood if proper postnatal infant care is implemented for the infants to reach their potential [30, 31]. In some cases [22], young maternal age and infant growth is linked through epigenetic effects. At the same time however, socioeconomic and environmental differences affect optimal linear growth of infants. A large birth cohort study [29] indicated the role of socioeconomic inequalities rather than maternal youth per se to be important factors to gain greater population benefits while promoting for better health outcomes of children from young mothers. The mothers involved in the present study were not significantly different by most of their socio-economic and nutritional characteristics irrespective of their age (Table 1). This buffers the association of maternal youth with infant growth, as the linear growth will not be different among the groups to be explained by variations in maternal socio-economic and nutritional characteristics.

Our analysis also revealed that, illness of the infant during the follow-up period reduced the linear growth significantly. Environmental exposures leading to infections during early childhood pose a major threat to optimal childhood growth through disrupting child feeding and caring practices [32]. Similarly the effects of mother’s poor nutritional, SES and child caring practices are more prominent among adolescent pregnancies putting their infants at risk of infections. The biological mechanism [8, 13, 29] by which, competing developmental process in these mothers could also have resulted in fetal developmental deficits which could later expose the infants for repeated infections.

### Implication

Child undernutrition takes significant share in causing deaths among under five children worldwide [6, 33] and poses serious health, social and economic threats throughout the life course [34]. Studies reported that stunting during early childhood is linked to impaired cognitive development, poor school achievement and decreased economic productivity in later life [34]. Women who are stunted are also likely to face adverse reproductive and maternal outcomes [18] continuing undernutrition through generations. About 90% of stunted children live in 36 African and Asian countries [33]. On the same note, child malnutrition is a persistent challenge in Ethiopia. Though stunting has decreased over time, 40% of under-five children yet remain stunted [35] causing severe social and economic costs. According to one report, the total losses associated with child undernutrition were estimated to be equivalent with 16.5% of the GDP [36]. Some initiatives and actions under the National Nutrition Program (NNP) have been rolled out widely and improved the nutritional status of infants, youth and children under five. Yet, low rates of implementation to improve nutritional status of women and adolescents have been reported [37]. Child bearing starts at early ages among Ethiopian women with 12% of adolescent girls being pregnant or gave birth to their first child already [38]. In such settings, implementing interventions targeting adolescent girls widely helps to break the intergenerational cycle of undernutrition. The strength of this study is the use of longitudinal data with a large sample size to show the association of young maternal age with linear growth of infants. On the other hand, the restriction of the follow up period only until 12 months can be mentioned as a limitation since we may not be able to capture the full spectrum of growth trajectories.

## Conclusion

We observed that young maternal age had a significant negative association with LAZ score of infants at birth while its association over time was not influential on the linear growth of infants. Furthermore, infant illness was associated with linear growth faltering. The fact that wide spread socio economic and environmental inequalities exist among mothers of all ages may have contributed to the non-significant association between young maternal age and linear growth faltering of infants. This leaves an opportunity to develop comprehensive interventions targeting for the infants to attain optimal catch-up growth.

## Additional files

Additional file 1: STROBE-nut check list. (DOCX 66 kb)

Additional file 2: Estimates table for the linear, quadratic and cubic-spline models. (DOCX 16 kb)

## Abbreviations

AIC: Akaike Information Criterion
BIC: Bayesian Information Criterion
ENGINE: Empowering the New Generation In Nutrition and Economic opportunities
LAZ: Length for Age Z-score
LMIC: Low and Middle Income Countries
