# Supplementary material for: Adolescent pregnancy and linear growth of infants: a birth cohort study in rural Ethiopia
Source: Nutr J. 2019 Apr 2;18:22. doi: 10.1186/s12937-019-0448-0 (PMC6806577; doi:10.1186/s12937-019-0448-0)
Supplement: Supplementary file 1 — STROBE-nut check list. (DOCX 66 kb) [file 12937_2019_448_MOESM1_ESM.docx]

**Additional file 3:** STROBE-nut: An extension of the STROBE statement for nutritional epidemiology


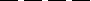


Lachat C et al. (2016) STrengthening the Reporting of OBservational studies in Epidemiology – Nutritional Epidemiology (STROBE-nut): an extension of the STROBE statement. Plos Medicine 13(6) http://dx.doi.org/10.1371/journal.pmed.1002036 pdf or online version.

|  | **Item** | **Item** | **STROBE recommendations** | **Extension for Nutritional** | Reported on page # |  |
| --- | --- | --- | --- | --- | --- | --- |
|  |  | **nr** |  | **Epidemiology studies** |  |  |
|  |  |  |  | **(STROBE-nut)** |  |  |
|  |  |  |  |  |  |  |
|  | **Title and** | 1 | (a) Indicate the study’s design with a | **nut-1** State the dietary/nutritional | Title p.1; Abstract p.2-3 |  |
|  | **abstract** |  | commonly used term in the title or the | assessment method(s) used in the |  |  |
|  |  |  | abstract. | title, abstract, or keywords. |  |  |
|  |  |  |  |  |  |  |
|  |  |  | (b) Provide in the abstract an |  |  |  |
|  |  |  | informative and balanced summary of |  |  |  |
|  |  |  | what was done and what was found. |  |  |  |
|  |  |  |  |  |  |  |
|  | **Introduction** |  |  |  |  |  |
|  |  |  |  |  |  |  |
|  | Background | 2 | Explain the scientific background and |  | Background p.4-5 |  |
|  | rationale |  | rationale for the investigation being |  |  |  |
|  |  |  | reported. |  |  |  |
|  | Objectives | 3 | State specific objectives, including any |  | Background p.5 last statements |  |
|  |  |  | pre-specified hypotheses. |  |  |  |
|  |  |  |  |  |  |  |
|  | **Methods** |  |  |  |  |  |
|  |  |  |  |  |  |  |
|  | Study design | 4 | Present key elements of study design |  | Methods (data source) p.5-6 |  |
|  |  |  | early in the paper. |  |  |  |
|  |  |  |  |  |  |  |


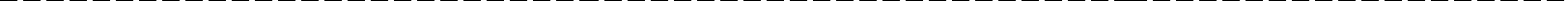

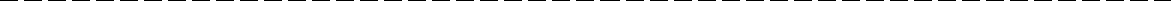


1

| **Item** | **Item** | **STROBE recommendations** | **Extension for Nutritional** | Reported on page # |
| --- | --- | --- | --- | --- |
|  | **nr** |  | **Epidemiology studies** |  |
|  |  |  | **(STROBE-nut)** |  |
|  |  |  |  |  |
| Settings | 5 | Describe the setting, locations, and | **nut-5** Describe any characteristics of | Methods (data source) p.5-6 |
|  |  | relevant dates, including periods of | the study settings that might affect |  |
|  |  | recruitment, exposure, follow-up, and | the dietary intake or nutritional |  |
|  |  | data collection. | status of the participants, if |  |
|  |  |  | applicable. |  |


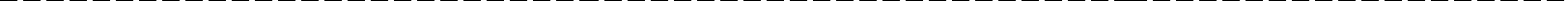

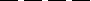


| Participants | 6 | a) Cohort study—Give the eligibility |
| --- | --- | --- |
|  |  | criteria, and the sources and methods |

of selection of participants. Describe

methods of follow-up.

Case-control study—Give the eligibility criteria, and the sources and methods of case ascertainment and control selection. Give the rationale for the choice of cases and controls.

Cross-sectional study—Give the eligibility criteria, and the sources and methods of selection of participants.

(b) Cohort study—For matched

studies, give matching criteria and

number of exposed and unexposed.

Case-control study—For matched studies, give matching criteria and the number of controls per case.


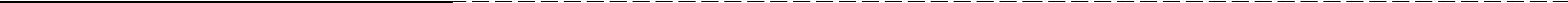


**nut-6** Report particular dietary, Methods (data source) p.5-6

physiological or nutritional characteristics that were considered when selecting the target population.

2

| **Item** | **Item STROBE recommendations** | **Extension for Nutritional** | Reported on page # |
| --- | --- | --- | --- |
|  | **nr** | **Epidemiology studies** |  |
|  |  | **(STROBE-nut)** |  |


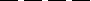


| Variables | 7 | Clearly define all outcomes, exposures, |
| --- | --- | --- |
|  |  | predictors, potential confounders, and |

effect modifiers. Give diagnostic

criteria, if applicable.


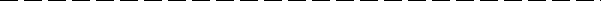


**nut-7.1** Clearly define foods, food groups, nutrients, or other food components.

**nut-7.2** When using dietary patterns or indices, describe the methods to obtain them and their nutritional properties.


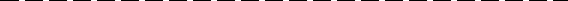


Methods (all outcome and exposure variables are listed) p.6-7


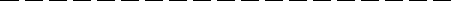


| Data sources - | 8 | For each variable of interest, give |
| --- | --- | --- |
| measurements |  | sources of data and details of methods |
|  |  | of assessment |
|  |  | (measurement).Describe |

comparability of assessment methods if there is more than one group.


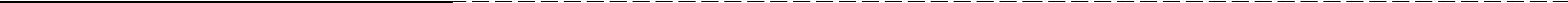


**nut-8.1** Describe the dietary assessment method(s), e.g., portion size estimation, number of days and items recorded, how it was developed and administered, and how quality was assured. Report if and how supplement intake was assessed.

**nut-8.2** Describe and justify food composition data used. Explain the procedure to match food composition with consumption data. Describe the use of conversion factors, if applicable.

**nut-8.3** Describe the nutrient requirements, recommendations, or dietary guidelines and the

Methods (methods of measurement indicated in) p.6-7

3

| **Item** | **Item STROBE recommendations** | **Extension for Nutritional** | Reported on page # |
| --- | --- | --- | --- |
|  | **nr** | **Epidemiology studies** |  |
|  |  | **(STROBE-nut)** |  |


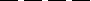


| Bias | 9 | Describe any efforts to address |
| --- | --- | --- |
|  |  | potential sources of bias. |
|  |  |  |


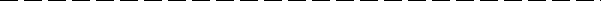


evaluation approach used to

compare intake with the dietary

reference values, if applicable.

**nut-8.4** When using nutritional

biomarkers, additionally use the

STROBE Extension for Molecular

Epidemiology (STROBE-ME).

Report the type of biomarkers used

and their usefulness as dietary

exposure markers.

**nut-8.5** Describe the assessment of

nondietary data (e.g., nutritional

status and influencing factors) and

timing of the assessment of these

variables in relation to dietary

assessment.

**nut-8.6** Report on the validity of

the dietary or nutritional

assessment methods and any

internal or external validation used

in the study, if applicable.


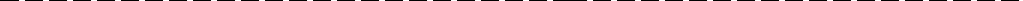


**nut-9** Report how bias in dietary or Methods (data quality methods

nutritional assessment was indicated) p.7

addressed, e.g., misreporting,


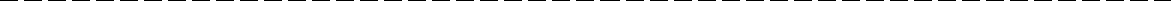


4

| **Item** | **Item STROBE recommendations** | **Extension for Nutritional** | Reported on page # |
| --- | --- | --- | --- |
|  | **nr** | **Epidemiology studies** |  |
|  |  | **(STROBE-nut)** |  |


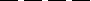


| Study Size | 10 | Explain how the study size was arrived |
| --- | --- | --- |
|  |  | at. |


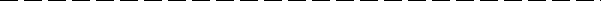

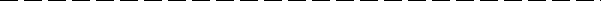


changes in habits as a result of being measured, or data imputation from other sources


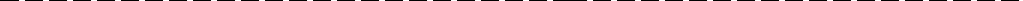


Methods (data source,

statistical analysis) p.5-6


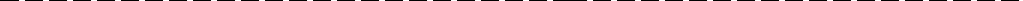


| Quantitative | 11 | Explain how quantitative variables |
| --- | --- | --- |
| variables |  | were handled in the analyses. If |
|  |  | applicable, describe which groupings |
|  |  | were chosen and why. |


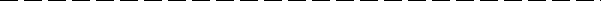


**nut-11** Explain categorization of dietary/nutritional data (e.g., use of N-tiles and handling of nonconsumers) and the choice of reference category, if applicable.


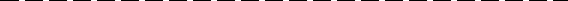


Methods (measurement and statistical analysis sections) p. 6, 7, 8


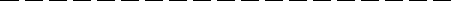


| Statistical | 12 | (a) Describe all statistical methods, |
| --- | --- | --- |
|  |  | including those used to control for |

| Methods | confounding |  |
| --- | --- | --- |
|  |  |  |
|  | (b) Describe any methods used to |  |
|  | examine subgroups and interactions. |  |
|  | (c) Explain how missing data were |  |
|  | addressed. |  |
|  | (d) Cohort study—If applicable, |  |
|  | explain how loss to follow-up was |  |
|  | addressed. |  |
|  |  |  |

**nut-12.1** Describe any statistical method used to combine dietary or nutritional data, if applicable.

**nut-12.2** Describe and justify the method for energy adjustments, intake modeling, and use of weighting factors, if applicable.

**nut-12.3** Report any adjustments for measurement error, i.e,. from a validity or calibration study.


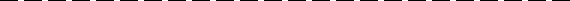


Methods (analysis section) p. 8

5

| **Item** | **Item STROBE recommendations** | **Extension for Nutritional** | Reported on page # |
| --- | --- | --- | --- |
|  | **nr** | **Epidemiology studies** |  |
|  |  | **(STROBE-nut)** |  |


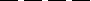


Case-control study—If applicable,

explain how matching of cases and

controls was addressed.

Cross-sectional study—If applicable,

describe analytical methods taking

account of sampling strategy.

(e) Describe any sensitivity analyses.

**Results**

| Participants | 13 | (a) Report the numbers of individuals |
| --- | --- | --- |
|  |  | at each stage of the study—e.g., |
|  |  | numbers potentially eligible, examined |
|  |  | for eligibility, confirmed eligible, |
|  |  | included in the study, completing |
|  |  | follow-up, and analyzed. |
|  |  | (b) Give reasons for non-participation |
|  |  | at each stage. |
|  |  | (c) Consider use of a flow diagram. |
| Descriptive data | 14 | (a) Give characteristics of study |
|  |  | participants (e.g., demographic, |


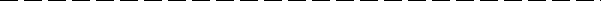


clinical, social) and information on

exposures and potential confounders


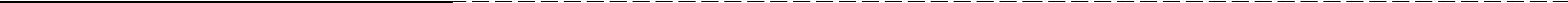


**nut-13** Report the number of individuals excluded based on missing, incomplete or implausible dietary/nutritional data.


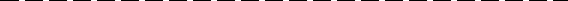


**nut-14** Give the distribution of participant characteristics across the exposure variables if applicable. Specify if food consumption of total

Results (result section, flow diagram included) p. 9-10


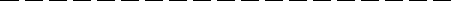


Results (table 1)p.11

6

| **Item** | **Item STROBE recommendations** | **Extension for Nutritional** | Reported on page # |
| --- | --- | --- | --- |
|  | **nr** | **Epidemiology studies** |  |
|  |  | **(STROBE-nut)** |  |


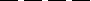


|  |  | (b) Indicate the number of |
| --- | --- | --- |
|  |  | participants with missing data for each |
|  |  | variable of interest |
|  |  | (c) Cohort study—Summarize follow- |
|  |  | up time (e.g., average and total |
|  |  | amount) |
| Outcome data | 15 | Cohort study—Report numbers of |
|  |  | outcome events or summary measures |
|  |  | over time. |
|  |  | Case-control study—Report numbers |
|  |  | in each exposure category, or |
|  |  | summary measures of exposure. |
|  |  | Cross-sectional study—Report |
|  |  | numbers of outcome events or |
|  |  | summary measures. |
| Main results | 16 | (a) Give unadjusted estimates and, if |
|  |  | applicable, confounder-adjusted |


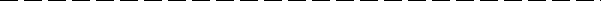

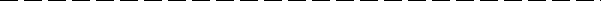


estimates and their precision (e.g.,

95% confidence interval).

Make clear which confounders were

adjusted for and why they were

included.


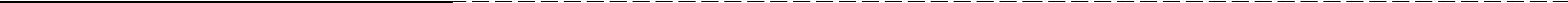


population or consumers only were used to obtain results.


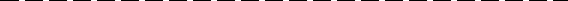

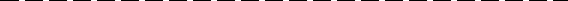


**nut-16** Specify if nutrient intakes are reported with or without inclusion of dietary supplement intake, if applicable.


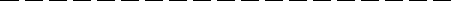


p. 9, 10, p.11 table 1

Results p.12-13, Table 2


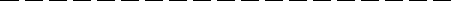


Results (p.12-13, table 2 )

7

| **Item** | **Item** | **STROBE recommendations** | **Extension for Nutritional** | Reported on page # |
| --- | --- | --- | --- | --- |
|  | **nr** |  | **Epidemiology studies** |  |
|  |  |  | **(STROBE-nut)** |  |
|  |  |  |  |  |
|  |  | (b) Report category boundaries when |  |  |
|  |  | continuous variables were categorized. |  |  |
|  |  | (c) If relevant, consider translating |  |  |
|  |  | estimates of relative risk into absolute |  |  |
|  |  | risk for a meaningful time period. |  |  |
| Other analyses | 17 | Report other analyses done—e.g., | **nut-17** Report any sensitivity | No additional analyses |
|  |  | analyses of subgroups and interactions | analysis (e.g., exclusion of | conducted |
|  |  | and sensitivity analyses. | misreporters or outliers) and data |  |
|  |  |  | imputation, if applicable. |  |
|  |  |  |  |  |
| **Discussion** |  |  |  |  |


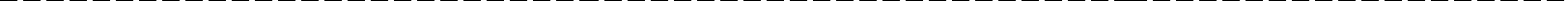

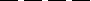


| Key results | 18 | Summarize key results with reference |
| --- | --- | --- |
|  |  | to study objectives. |
| Limitation | 19 | Discuss limitations of the study, taking |
|  |  | into account sources of potential bias |
|  |  | or imprecision. Discuss both direction |
|  |  | and magnitude of any potential bias. |


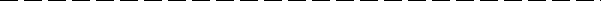

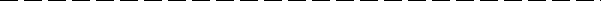


**nut-19** Describe the main limitations of the data sources and assessment methods used and implications for the interpretation of the findings.


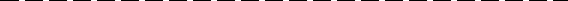

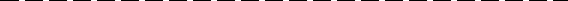


Discussion (statement of principal findings) p.14-16


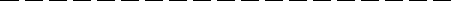


Discussion (strengths and weaknesses of the study) p.16 before conclusion


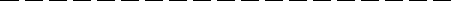


| Interpretation | 20 | Give a cautious overall interpretation |
| --- | --- | --- |
|  |  | of results considering objectives, |
|  |  | limitations, multiplicity of analyses, |

results from similar studies, and other relevant evidence.


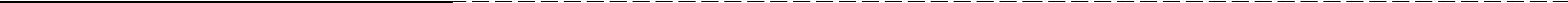


**nut-20** Report the nutritional relevance of the findings, given the complexity of diet or nutrition as an exposure.

Discussion (interpretation of findings in the context of existing research, meaning of the study:

p.15-16 implication

8

| **Item** | **Item** | **STROBE recommendations** | **Extension for Nutritional** | Reported on page # |
| --- | --- | --- | --- | --- |
|  | **nr** |  | **Epidemiology studies** |  |
|  |  |  | **(STROBE-nut)** |  |
|  |  |  |  |  |
|  |  |  |  |  |
|  |  |  |  |  |
| Generalizability | 21 | Discuss the generalizability (external |  | Discussion (strengths and |
|  |  | validity) of the study results. |  | weaknesses of the study) |
|  |  |  |  | p.16 |
|  |  |  |  |  |
| **Other information** |  |  |  |  |
|  |  |  |  |  |
| Funding | 22 | Give the source of funding and the role |  | Funding p.17 |
|  |  | of the funders for the present study |  |  |
|  |  | and, if applicable, for the original |  |  |
|  |  | study on which the present article is |  |  |
|  |  | based. |  |  |
| *Ethics* |  |  | **nut-22.1** Describe the procedure | p.8 and 17 |
|  |  |  | for consent and study approval from |  |
|  |  |  | ethics committee(s). |  |


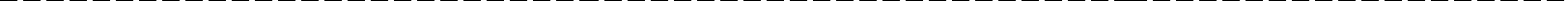

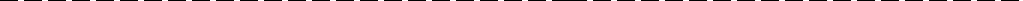

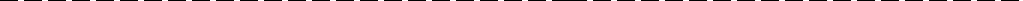


*Supplementary* **nut-22.2** Provide data collection

*material* tools and data as online material or

explain how they can be accessed.

p.17

9
