# Supplementary material for: Adolescent pregnancy and linear growth of infants: a birth cohort study in rural Ethiopia
Source: Nutr J. 2019 Apr 2;18:22. doi: 10.1186/s12937-019-0448-0 (PMC6806577; doi:10.1186/s12937-019-0448-0)
Supplement: Supplementary file 2 — Estimates table for the linear, quadratic and cubic-spline models. (DOCX 16 kb) [file 12937_2019_448_MOESM2_ESM.docx]

| Variables | **Coefficient (SE)** linear model | **Coefficient (SE)** Quadratic model | **Coefficient (SE)**  Cubic spline model |
| --- | --- | --- | --- |
| Constant | .98 | 1.03 | -.14 |
| Infant age (time) | .06 (.023)$ | .14 (0 .03)$ | - |
| Quadratic time effect (Time*Time) | - | -.01 | - |
| Maternal age(15-19) | -.14 (.091) | -.24 (0 .13)$ | 1.44 (1.29) |
| Maternal age*Time | .018 (.008) | 0.05 (0.034) | - |
| LAZbaseline | .24 (.016)$ | 0.38 (0.029)$ | .41 (.030)$ |
| Maternal education  (ref=no formal education) |  |  |  |
| Primary | .06 (.078) | .05 (0.077) | .05 (.077) |
| Primary*Time | .01 (.007) | .020 (0.007) | .014 (.007) |
| ≥Secondary | .13 (.136) | .10 (0.135) | .10 (.134) |
| ≥Secondary*Time | .03 (.013) | .03 (0.013)$ | .03 (.013)$ |
| Wealth index (ref=low) |  |  |  |
| Middle | .23 (.085)$ | .21(0.084) | .20 (.084)$ |
| Middle*Time | .01 (.008) | 0.02 (0.008) | .01 (.008) |
| High | .20 (.085)$ | .13 (0.084) | .11 (.084)$ |
| High*Time | .004 (.008) | .01 (0.008) | .001 (.008) |
| Iron-folate supplement (≥90 days) | -.07 (.075) | -.07 (0.074) | -.08 (.073) |
| Iron-folate*Time | .015 (.007)$ | .015 (0.007) | .016 (.007)$ |
| Illness in 2 weeks (Yes) | .06 (.071) | .05 (0.070) | .04 (.070) |
| Illness in 2 weeks *Time | -.10 (.006)$ | -.15 (0.006) | -.015 (.006)$ |
| Age_y1 |  |  | .79 (.278)$ |
| Age_y2 |  |  | -2.40 (.984)$ |
| Age_y3 |  |  | 4.66 (2.17)$ |
| Age_y4 |  |  | -2.20 (1.52) |
| Maternal age* Age_y1 |  |  | -.63 (.532) |
| Maternal age* Age_y2 |  |  | 2.22 (1.89) |
| Maternal age* Age_y3 |  |  | -4.70 (4.20) |
| Maternal age* Age_y4 |  |  | 2.65 (2.94) |
| Constant | .98 | 1.38 | -.14 |
| **Statistics** |  |  |  |
| **Number of groups (N)** | **1,378** | **1,378** | **1,378** |
| **AIC** | **13340.397** | **13131.962** | **13099.42** |
| **BIC** | **13597.659** | **13402.329** | **13495.997** |

**Estimates table for the linear, quadratic and cubic spline models**

**SE:** standard error**, $:**significance level at p<0.05**, AIC:** Akaike Information Criterion**, BIC:** Bayesian Information Criterion
